# The Collapse of the Price of Oil and the Importance of Fair Market Competition and Optimizing Public and Private Resources: Assessing Angola's Contraceptive Market Landscape

**DOI:** 10.9745/GHSP-D-17-00165

**Published:** 2017-09-27

**Authors:** Denise L Harrison

**Affiliations:** a U.S. Agency for International Development, Arlington, VA, USA.

See related article by Nieto-Andrade et al. and authors' response.

In GHSP's March 2017 article by Nieto-Andrade and colleagues, “Women's Limited Choice and Availability of Modern Contraception at Retail Outlets and Public-Sector Facilities in Luanda, Angola, 2012–2015,” the authors assert that public health policies must ensure the availability and affordability of contraceptives on the market and expand the range of options for women.^1^ I would argue that public health policies should instead support fair market competition and optimize the use of both public and private resources. If subsidies are necessary, they should be discrete, targeted, and time-bound to reduce crowding out the commercial private sector, which increases the cost of family planning for donors and governments (i.e., taxpayers). If the authors had provided more information about Angola's economic crisis, shared a deeper discussion around market competitiveness, and disclosed their own plans to launch both oral and emergency contraceptive pills in Angola, GHSP readers would have obtained a better understanding of Angola's contraceptive market landscape, as well as the interest of the authors as market players.

## ANGOLA'S ECONOMIC CRISIS

In 2014–15, oil prices fell by nearly 50%. Annual inflation doubled from 7.5% in 2014 to 15.3% in 2015, and the Angolan Kwanza currency declined by more than 35% against the dollar.^2^^,^^3^ Prices for pharmaceuticals (not just contraceptives), which are mainly imported, jumped. Exacerbating this situation was the Bank of America and Standard Chartered Bank's decision to stop supplying dollars to Angola based on their concern about lax banking regulations.

The authors state “the availability of different methods dropped significantly between 2014 and 2015—by up to 15 percentage points—with a subsequent price increase in many brands,” linking the increase in contraceptive prices to the decline in method availability. However, it was the weak economy, rampant inflation, and a shortage of foreign exchange, which led to a steep decline in imports, that caused both the price increases and the decline in method availability. Since 2013, I have been visiting Luanda for work periodically and have seen firsthand the closing of many pharmacies starting in 2014. It was clear that the shortage of foreign exchange was causing pharmacies to go out of business.

The weak economy, rampant inflation, and a shortage of foreign exchange caused both the contraceptive price increases and declines in method availability in Angola.

## MEASURING MARKET COMPETITIVENESS

The article by Nieto-Andrade et al. asserts there is limited choice and availability of contraceptives in Angola, pointing out that 85.9% of all outlets had only 1 method of contraception in 2014 (81.4% in 2015). These statistics are not strong evidence of limited choice and availability in a country where the modern contraceptive prevalence rate (mCPR) is under 20%.^4^ Nigeria, like Angola, is also middle income, has an economy dependent on oil revenues, and an mCPR under 20%.^5^^,^^6^ In 2015, Population Services International (PSI) conducted an “FPwatch” study in Nigeria, which found that only 2,553 of the 13,365 outlets surveyed carried condoms—let alone hormonal contraceptives.^7^ “Limited” is contextual; a richer analysis is needed to support the title's assertion.

The authors state “the number of brands on the market is an indicator of the number of choices available to women in terms of quality and prices.” This is only true where the number and size of manufacturers on the market is competitive. Bayer manufactures numerous oral contraceptive brands, most of which are available in Angola. Bayer contraceptives dominate Angola's market even when leaked donated Bayer contraceptives are taken out of the equation.^8^ Manufacturer (or distributor) concentration can reduce family planning choice, availability, and affordability. Tracking manufacturer presence, entry, and exit is a more appropriate way to understand market health, growth potential, and consumer choice than tracking only the number of brands.^9^

The same assessment of market dynamics to determine market health applies to the condom market, as well. Because socially marketed condoms receive direct and indirect subsidies, to gauge market health, it is important to understand how well these condoms are targeted, their pricing, and their impact on current and future market growth and consumer choice. The article states “socially marketed, affordably priced condoms are successfully competing as indicated by their high market share in terms of both volume and value …” Legal and Sensual condoms distributed by PSI (the authors' affiliation) are highly subsidized either through a price subsidy, tax-free status, and/or support for advertising and operations. PSI has a cost advantage over other distributors, which contributes to its domination of the market. As a subsidized social marketing organization, PSI should have underscored its success in reaching its target population, changing their behaviors, and “crowding in” quality manufacturers, and explaining at what point subsidies could be phased out.

## CLARITY ON PSI'S OWN INVOLVEMENT IN THE MARKET

Finally, for a deeper understanding of the market landscape, it is helpful to know all the ways in which the authors are involved in the market. With funding from the Bill & Melinda Gates Foundation, PSI is entering the hormonal contraceptive market in Angola with an oral contraceptive pill and an emergency contraceptive, a fact that was not disclosed in the article. Is this why the article says women have “limited choice” and emphasizes the importance of “preventing leakage of ‘free’ products from the public sector into the private sector (which are later sold at ‘unfair’ prices)” to ensure the private sector invests more? The price for leaked Microgynon oral contraceptive pills in Angola is around US$2.50, higher than many—if not most—PSI socially marketed oral contraceptives in sub-Saharan Africa. Is it PSI's goal to succeed by capturing a large part of the hormonal contraceptive market in addition to the condom market? What percentage of these subsidized sales will reach populations at low quintiles, “crowd out” commercial players, or raise the mCPR and lower the total fertility rate?

Leakage, market concentration, and overly or poorly controlled subsidization can impede market health and growth, reducing women's choices for family planning today and tomorrow. The authors rightly point out the new Angolan context, in which donors are reducing donation of products and focusing mostly on technical assistance, requires good public health policies. It also requires transparency and good will on everyone's part, including NGOs as well as governments and donors.

Leakage, market concentration, and overly or poorly controlled subsidization can impede market health and growth.
